# Flying fish as useful model for evolutionary investigations in epipelagic zones. A cytogenomic and cytotaxonomic approach

**DOI:** 10.3897/compcytogen.20.190559

**Published:** 2026-06-12

**Authors:** Gideão Wagner Werneck Felix da Costa, Oscar Akio Shibatta, Marcelo de Bello Cioffi, Jhon Alex Dziechciarz Vidal, Luiz Antonio Carlos Bertollo, Wagner Franco Molina

**Affiliations:** 1 Department of Cell Biology and Genetics, Biosciences Center, Federal University of Rio Grande do Norte, 59078970, Natal, RN, Brazil Department of Genetics and Evolution, Federal University of São Carlos São Carlos Brazil; 2 Department of Animal and Plant Biology, Biological Science Center, State University of Londrina, 86051-970 - Londrina, PR, Brazil Department of Cell Biology and Genetics, Biosciences Center, Federal University of Rio Grande do Norte Natal Brazil; 3 Department of Genetics and Evolution, Federal University of São Carlos, Rod. Washington Luis, km 235, 13565-905 São Carlos, SP, Brazil Department of Animal and Plant Biology, Biological Science Center, State University of Londrina Londrina Brazil

**Keywords:** Chromosomal evolution, fish cytogenetics, Marine fish, repetitive sequences

## Abstract

Flying fishes (Exocoetidae) constitute a group of fish with adaptations for life in the epipelagic zone, notably featuring one or two pairs of expanded fins that enable gliding flight. Most of their 80 species have an interoceanic distribution, where they stand out as economic resources, as well as a key component of the trophic base of large pelagic predators. However, despite being a charismatic evolutionary model with considerable knowledge about its biology, that group remains neglected regarding its cytogenetic characterization. Here, we performed cytogenetic analyses for the first time in *Hirundichthys affinis* (Günther, 1866) (two Atlantic populations) and in *Cheilopogon exsiliens* (Linnaeus, 1771) and *C. furcatus* (Mitchill, 1815) (Saint Peter and Saint Paul Archipelago, mid-Atlantic region). These analyses included C-banding, silver nitrate staining (Ag-NOR), and fluorescent *in situ* hybridization (FISH) with 18S rDNA, 5S rDNA, and (CA)_15_ microsatellite probes. The three species share 2n = 48 but exhibit discernible intergeneric karyotypic divergences. Thus, while the karyotype of *H. affinis* displays exclusively acrocentric chromosomes (48a, FN = 48), *C. exsiliens* (2st+46a, FN = 50) and *C. furcatus* (2m+46a, FN = 50) both differ due to one pair of bi-armed chromosomes. Heterochromatin shows an occasional accumulation of (CA)_15_ clusters predominantly in pericentromeric regions of the chromosomes. The rDNA sites display variations in number and location, proving to be effective cytotaxonomic and population markers for the group. Our data indicate that the distribution of the flying fishes was accompanied by their chromosomal reorganizations. Thus, cytogenetic data stand out as promising tools for unraveling environmental adaptations, reproductive isolation, and speciation in the vast epipelagic areas they occupy.

## Introduction

The epipelagic zones of the Pacific, Indian, and Atlantic Oceans constitute the largest ecosystem on the planet, harboring an abundant and unique fauna (Flegontova et al. 2016), where the charismatic Exocoetidae fish family, commonly known as flying fish, stands out (Pierucci and Suaria 2023). In fact, its representatives have extremely elongated and rigid pectoral fins (Davenport 1994, 2003), which enable them to glide above the water’s surface, a remarkable behavioral adaptation against predators (Davenport 1994; Parin and Shakhovskoy 2000; Lewallen et al. 2017). In addition, they also exhibit other adaptations to the ocean surface, such as vision both in and out of water (Davenport 1994; Reckel and Melzer 2004); buoyant eggs or those attached to filamentous structures (Kovalevskaya 1982; Collette et al. 1984; Gordeeva and Shakhovskoi 2017); and body fat providing increased buoyancy in the water column (Blake 1983; Pelster 2009).

Beyond their socioeconomic relevance, due to their roe commanding a high market price and the dried fish being used for local subsistence (Araújo and Chellappa 2002), flying fish play a key ecological role by converting energy from phytoplankton into biomass accessible to higher trophic levels, serving as prey for marine mammals, seabirds, and commercially valuable predatory fish (Araújo et al. 2011; Nelson et al. 2016).

Exocoetidae are a monophyletic group (Lewallen et al. 2011), including four subfamilies (Fodiatorinae, Parexocoetinae, Exocoetinae, and Cypselurinae), and comprising seven genera and 80 species (Fricke et al. 2025). Most species have broad distributions across the major ocean basins (Parin 1961; Collette et al. 1984). The coexistence of widely and restricted distributed species makes Exocoetidae a particular model for investigating patterns of population structure across vast oceanic scales (Chou et al. 2015; Lewallen et al. 2016; Indrayani et al. 2024).

Although flying fish have been relatively well studied regarding their biology (Herawati et al. 2005; Araújo et al. 2011; Van Noord et al. 2013; Tuapetel et al. 2017), ecology (Randall et al. 2015; Lewallen et al. 2018; Pierucci and Suaria 2023), and genetics (Lewallen et al. 2011, 2017; Gordeeva and Shakhovskoi 2017; Ding et al. 2023, Silva et al. 2025), information on their cytogenetics remains scarce (Arai 2011; Molina et al. 2024b).

Karyotypic analyses of marine fish have provided important information regarding their life history, such as ecogeographic variations (Lima-Filho et al. 2012; Ghigliotti et al. 2020), population polymorphisms (Getlekha et al. 2016), evolutionary trends (Molina et al. 2014a, 2014b, 2023, 2024a; Romeiro et al. 2025), sex chromosome differentiation (Soares et al. 2014), genome size (Neafsey and Palumbi 2003; Hardie and Hebert 2004; Comber and Smith 2004) and reproductive isolation (Molina et al. 2024a). In this context, given their notable sympatric overlap, chromosomal analyses of flying fish species can provide important insights into understanding reproductive isolation between species.

So, here we conducted the first cytogenetic analyses of three flying fish species from two Atlantic populations, including their C-banding patterns, Ag-NOR distributions, and mapping of repetitive sequences through fluorescent *in situ* hybridization (FISH). The results highlighted specific chromosomal reorganizations, as well as conspicuous interpopulational variations. The data are relevant for evaluating taxonomic questions, detecting cryptic lineages and investigating incipient diversification processes within the group.

## Material and methods

### Sampling and chromosomal preparations

**Figure 1.**
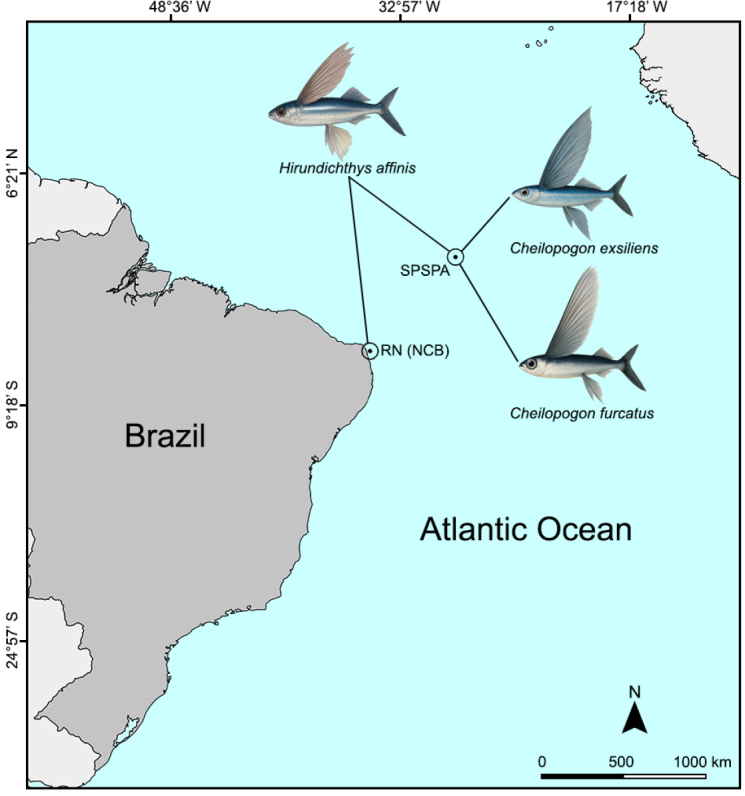
Collection sites of Exocoetidae flying fish species. RN (NCB) = Caiçara do Norte, Northeast Coast of Brazil; SPSPA = São Pedro and São Paulo Archipelago.

Samples of *Hirundichthys affinis* (Günther, 1866) (Fourwing flyingfish) were from the São Pedro and São Paulo Archipelago (SPSPA: 0°55'00.2"N, 29°20'44.1"W; n = 15; 10 males and five females) and Caiçara do Norte on the Northeast Coast of Brazil (NCB: n = 30; 15 males and 15 females). Samples of *Cheilopogon exsiliens* (Linnaeus, 1771) (Bandwing flyingfish: n = 8) and *Cheilopogon furcatus* (Mitchill, 1815) (Spotfin flyingfish: n = 10) were also from the SPSPA, all of them of undetermined sex (Fig. 1). All collections and handling procedures were in accordance with the Animal Ethics Committee (CEUA) of the Federal University of Rio Grande do Norte (Protocol No. 44/2015). Vouchers of species from SPSPA are available at Zoology Museum of State University of Londrina (MZUEL), under catalog numbers MZUEL 20473 (*H. affinis*), MZUEL 20474 (*C. exsiliens*), and MZUEL 20476 (*C. furcatus*).

To obtain mitotic chromosomes, specimens were treated for 50–60 minutes with a 0.025% aqueous colchicine solution (1 mL/100 g of body weight), injected into the dorsal region and intraperitoneal cavity, before removing the anterior portion of the kidney (Bertollo et al. 1978). As an alternative procedure, anterior kidneys cells were also dissociated in RPMI 1640 culture medium, for a short-term *in vitro* culture (Gold et al. 1990). Chromosomal analyses included Giemsa staining, C-banding (Sumner 1972), silver nitrate impregnation (Ag-NOR; Howell and Black 1980), fluorescence *in situ* hybridization (FISH) using 18S rDNA, 5S rDNA, and (CA)_15_ microsatellite probes.

### Probes for chromosomal hybridization

The 18S rDNA and 5S rDNA probes were amplified by polymerase chain reaction (PCR) using the primers 18S F (5'-CCG CTT TGG TGA CTC TTG AT-3') and 18S R (5'-CCG AGG ACC TCA CTA AAC CA-3') (Gross et al. 2010), and 5S F (5'-TAC GCC CGA TCT CGT CCG ATC-3') and 5S R (5'-CAG GCT GGT ATG GCC GTA AGC-3') (Martins and Galetti Jr 1999), respectively. Following amplification, the 18S rDNA sequences were labeled via nick translation with Atto550 NT Labeling Kit fluorophores, and the 5S rDNA sequences with the Atto488 NT Labeling Kit (Jena Bioscience GmbH, Germany). The (CA)_15_ dinucleotide probes were directly labeled with the Cy5 fluorophore during synthesis (Sigma, St. Louis, MO, USA).

### Hybridization and chromosomal analysis

FISH experiments essentially followed the protocol of Pinkel et al. (1986). Slides with the cell suspension were treated with RNase (20 µg/ml in 2xSSC) at 37 °C for 1 hour, and with pepsin (0.005% in 10 mM HCl) for 10 minutes. Subsequently, they were immersed in 1% formaldehyde for 10 minutes, washed in 1x PBS for 5 minutes, and subjected to an alcohol series (70%/85%/100%) for 5 minutes at each step. After that, they were incubated and denatured in 70% formamide/2xSSC at 72 °C for 5 minutes and dehydrated again through the same alcohol series. The hybridization process was carried out in a solution of 50% formamide, 2xSSC, 10% dextran sulfate, and the denatured probe (5 ng/µl), in a final volume of 30 µl, for 16 hours at 37 °C. After hybridization, slides were subjected to a series of washes: in 1x SSC at 65 °C (30 minutes), 1xSSC (5 minutes), Tween 20: 0.5%/4xSSC (5 minutes), and 1xPBS (1 minute). Subsequently, the slides were dehydrated through an alcohol series and air-dried. Hybridization signals were detected as previously described, and chromosomes were counterstained with Vectashield/DAPI (1.5 µg/ml) (Vector, Burlingame, USA).

Five individuals per species or population were employed for the mapping of 18S rDNA, 5S rDNA, and (CA) microsatellite sequences. Best metaphases were captured using an Olympus BX51 epifluorescence microscope, equipped with an Olympus DP73 digital capture system and cellSens 1.7 software (Olympus Optical Co. Ltd., Tokyo, Japan). Chromosomes were classified as metacentric (m), submetacentric (sm), subtelocentric (st), and acrocentric (a) according to their arm ratio (Levan et al. 1964). The fundamental number (FN) was determined by considering acrocentric chromosomes as having only one arm and all other ones as having two arms.

## Results

**Figure 2.**
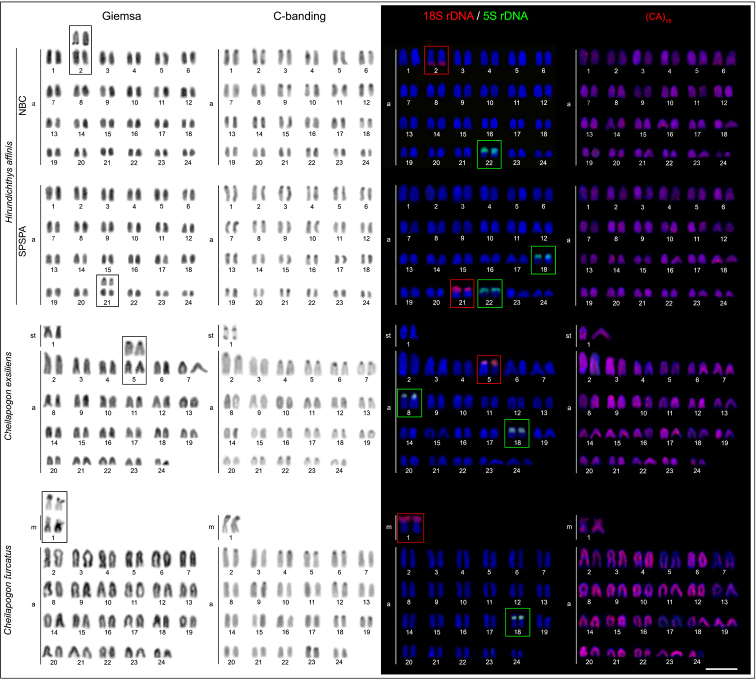
Karyotypes of *Hirundichthys affinis* (NCB: Northeast Coast of Brazil; SPSPA: São Pedro and São Paulo Archipelago), *Cheilopogon exsiliens* and *Cheilopogon furcatus* under Giemsa staining (Ag-NOR sites highlighted in boxes), C-banding, and FISH with 18S rDNA and 5S rDNA probes. Scale bar: 5 µm.

All species share the same diploid number, 2n = 48, but with divergences in the karyotypic structure. All analyzed individuals of *H. affinis* from both the SPSPA and NCB regions exhibited a karyotype composed exclusively of acrocentric chromosomes (2n = 48, FN = 48) (Fig. 1). On the other hand, *C. exsiliens* and *C. furcatus* have 2st+46a (FN = 50), and 2m+46a (FN = 50), respectively (Fig. 2).

The heterochromatin had a preferential location in the centromeric/pericentromeric regions of the chromosomes in all species, but also with some differential distribution among them. For instance, *H. affinis* individuals from the NCB region showed additional heterochromatic segments in the telomeric region of the long arms of pairs 2 and 14. In contrast, those from SPSPA displayed a heterochromatic segment in the interstitial region of the long arm of pair 3. In turn, the first chromosome pair 1 of *C. exsiliens* presents exclusive heterochromatic segments in both the centromeric region as well as in the telomeric region of the long arm. Furthermore, in *C. furcatus*, heterochromatin was localized in the centromeric regions and in the terminal region of the p arm of the first pair, which corresponds to the Ag-NOR region (Fig. 2)

Ag-NOR/18S rDNA sites occurred only in a single locus in all species, but in distinct chromosomal locations. In *H. affinis* from the NCB, they were in the terminal region of the long arm of pair 2, while in the population from SPSPA, they were situated in the terminal region of the short arms of pair 21. In *C. exsiliens* and *C. furcatus*, they were located in the short arm of pairs 5 and 1, respectively (Fig. 2). On the other hand, the 5S rDNA was distributed in one or two chromosomal loci. In *H. affinis* individuals from the NCB, it located in the pericentromeric region of pair 22, while in individuals from SPSPA it was found in the short arms of pairs 18 and 22. Different locations also occur between the two *Cheilopogon* species: in the short arms of pairs 8 and 18 of *C. exsiliens*, and in the short arm of pair 18 of *C. furcatus* (Fig. 2).

Across all examined species, *H. affinis* (from both NCB and SPSPA), *C. exsiliens*, and *C. furcatus*, the (CA)_15_ repeats exhibited a predominantly diffuse chromosomal distribution (Fig. 2).

## Discussion

All the flying fish species and populations analyzed share 2n = 48 chromosomes, a trait considered ancestral in Percomorpha (Galetti Jr et al. 2000; Motta-Neto et al. 2019). In addition, 2n = 48a (FN = 48), as occurs in *H. affinis*, is also an ancestral condition for the main groups of marine fish (Galetti Jr et al. 2000). In fact, the conservation of this basal chromosome number is notable in several marine fish groups (Motta-Neto et al. 2019; Amorim et al. 2017; Molina et al. 2024a), indicating a widespread karyotypic stasis (Molina 2007; Motta-Neto et al. 2011, 2019). This noteworthy feature likely encompasses biological, ecological, and environmental factors that promote gene flow (Molina 2007; Molina et al. 2014a, 2024a, 2024b). Among them, high dispersal potential covering vast interoceanic ranges, as well as large population sizes are common ones among flying fish.

Some pelagic marine fish exhibit an inverse correlation between their geographic distribution and karyotypic variation (Soares et al. 2021). In contrast, reef fish, which exhibit population structure throughout their range, are often more susceptible to fix conspicuous chromosomal rearrangements (Lima-Filho et al. 2012; Amorim et al. 2017; Nirchio et al. 2019), suggesting that restricted gene flow is a key driver of karyotypic divergence.

The three flying fish species, despite having a broad Atlantic distribution, showed clear evidence of structural karyotype divergence at the populational, intergeneric, and congeneric levels. In fact, it is known that some flying fish can display significant population structuring (Gordeeva and Shakhovskoi 2017). For example, clear *H. affinis* populations from the eastern Caribbean, southern Netherlands Antilles, and northeastern Brazil provided evidence for at least three distinct unit stocks in the central western Atlantic (Gomes et al. 1999). It is likely that such intraspecific genetic divergence in may extend beyond Central Atlantic populations of *H. affinis* and may be linked to conspicuous cytogenetic differences in the organization of their 18S and 5S rDNA sequences, as observed between populations from the Northeast Coast of Brazil (NCB) and the São Pedro and São Paulo Archipelago (SPSPA) in the meso-Atlantic region.

The conspicuous variation in rDNA site organization between the two *H. affinis* populations underscores the utility of rDNA sequence mapping as a chromosomal population marker. Indeed, a single 18S rDNA locus was in the long arm of the large acrocentric pair 2 of *H. affinis* individuals from SPSPA. In contrast, in NCB specimens it is situated on the short arm of the distinctly smaller acrocentric pair 21. Furthermore, 5S rDNA sites also differ between these populations in both frequency and chromosomal position, i.e., a single site on the small pair 22 of the NCB population, and two sites in the pairs 18 and 22 of the SPSPA population. The observed changes involving the differential organization of 18S rDNA sequences in *H. affinis* populations, associated with variable 5S rDNA arrays, are pronounced and can only be explained by a complex set of events, including transposition, translocation, and other chromosomal rearrangements. Notably, the level of rDNA variation surpasses that typically found in marine species. This finding not only confirms significant transatlantic population structure in *H. affinis* (Gomes et al. 1999) but also raises the possibility of cryptic speciation, a hypothesis that requires testing with molecular taxonomic approaches.

*Cheilopogon* Lowe, 1841 is a polyphyletic genus, emerging as the most diverse and morphologically variable among flying fish. It comprises two distinct clades, with *C. exsiliens* and *C. furcatus* belonging to a different one (Lewallen et al. 2011). The most distant evolutionary relationship between the two *Cheilopogon* species may explain the independent chromosomal rearrangements in each one of the lineages. In fact, *C. exsiliens* (2st+46a; FN = 50) and *C. furcatus* (2m+46a; FN = 50) revealed a discernible differentiation related to their first chromosome pair, which is subtelocentric in the former and metacentric in the latter species. This chromosome pair may have originated by pericentric inversions, a recognized driver of karyotypic diversification in marine Percomorpha (Galetti Jr et al. 2006; Amorim et al. 2017), or even by a centromere repositioning (Ansai et al. 2024), an evolutionary mechanism still poorly understood among fish, but which may explain some chromosomal changes in this group.

However, although the first pair was similar in size in both *Cheilopogon* species, it differs due to a terminal 18S rDNA/Ag-NOR site on the short arm in *C. furcatus*. Given the evolutionary lability of rDNA sites, these chromosomes may be homeologs ones that diverged through the loss or gain of a major rDNA site. This change also suggests underlying structural reorganization in the Ag-NOR-bearing chromosomal pairs.

The 5S rDNA sites also vary in number and position between *Cheilopogon* species. *Cheilopogon exsiliens* exhibits two loci, whereas *C. furcatus* possesses only one, each located on distinct chromosomal pairs. Indeed, ribosomal genes stand out as efficient chromosomal markers in flying fish and may be associated with interactions with other repetitive DNA elements, as well as the promotion of chromosomal rearrangements (Galetti Jr et al. 2000; Cioffi and Bertollo 2012; Gornung 2013; Amorim et al. 2017).

In general, flying fishes exhibited considerable diversification in rDNA sequence organization among populations, species, and genera. Despite the conserved diploid number, this numerical and structural diversification of rDNA suggests extensive internal chromosomal reorganization driven by a substantial set of rearrangements.

Heterochromatin constitutes a substantial fraction of fish genomes (Sun et al. 2020) and plays an important structural and functional role in their chromosomes (Cioffi and Bertollo 2012). Its distribution has also proven efficient in highlighting evolutionary differentiations among the flying fish species. Although primarily distributed in the pericentromeric regions, differentiations can be detected in relation to other chromosomal regions. In *H. affinis*, for example, heterochromatic sites were not only pericentromeric, but also interstitial and even with a bitelomeric distribution in some pairs. In turn, *C. exsiliens* displayed heterochromatin in both pericentromeric and interstitial regions on a few pairs, thus contrasting with the primarily pericentromeric distribution in *C. furcatus*.

**Figure 3.**
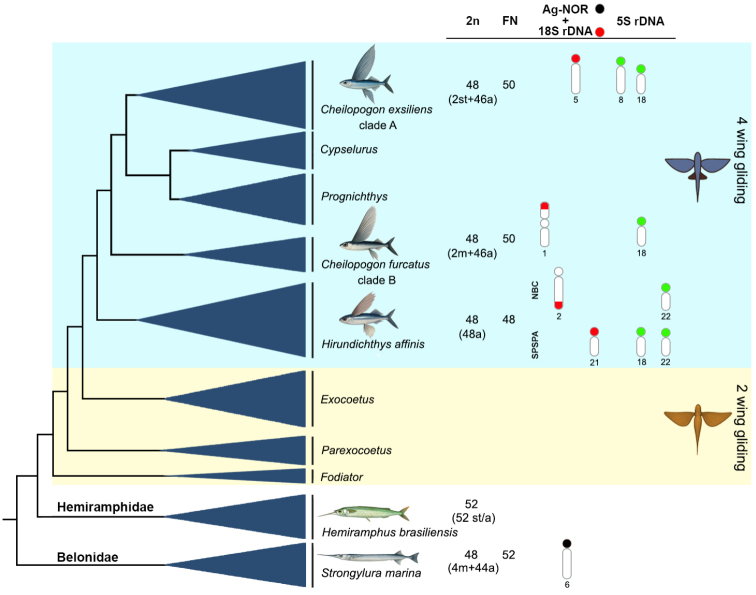
Phylogeny of Exocoetidae genera integrating molecular (Lewallen et al. 2011) and cytogenetic data (this contribution and Arai 2011), showing the evolutionary sequence of flight fin morphology.

Variation in the heterochromatin distribution may result in internal chromosomal rearrangements and the mobilization of repetitive elements and satellite sequences, as heterochromatin in fish chromosomes can harbor a variety of repetitive sequences, including microsatellites (Cioffi and Bertollo 2012; Costa et al. 2015; Silva et al. 2021; Romeiro et al. 2025). In all species analyzed, the (CA)_15_ sequences are primarily dispersed along the chromosome arms, a pattern also reported in other fish species (Shimoda et al. 1999; Cioffi et al. 2011; Xu et al. 2013; Supiwong et al. 2014). This suggests that dispersed microsatellite distribution is a common feature for this animal group (Fig. 3).

A broad assessment suggests that karyotypic evolution in flying fishes is more dynamic than expected for a transoceanic pelagic group, as revealed by chromosomal macrostructure and internal rDNA sequence divergences. The significant cytogenetic variation between *H. affinis* populations underscores a notably complex scenario of chromosomal diversification, contrasting with patterns seen in other pelagic fishes (Soares et al. 2013, 2021).

## Conclusion

Our current results reveal a set of chromosomal characteristics in the flying fish species, some conserved alongside others distinct. Differential features were found at interpopulation, interspecific, and congeneric levels, indicating greater evolutionary dynamism for this fish group than for other pelagic ones. The karyotypic differentiation between the two populations of *H. affinis*, spanning vast oceanic areas, is notable and can be explained by their pronounced population structure. The occurrence of a possible cryptic divergence in this group is not ruled out, and cytogenetic data will be valuable tools for future taxonomic investigations. Thus, flying fishes offer a promising model for studying speciation and evolutionary adaptation in the epipelagic zone, helping to address a significant gap in our understanding of the evolutionary dynamics within this key oceanic environment.

## Additional information

### Artificial Intelligence (AI) use

The authors accept full responsibility for the content of the manuscript, including the disclosure of any use of AI.

No AI tools were used in the preparation of this manuscript.

### Author ORCIDs

O.A. Shibatta https://orcid.org/0000-0003-2883-5934

M.B. Cioffi https://orcid.org/0000-0003-4340-1464

J.A.D. Vidal https://orcid.org/0000-0001-5779-0443

L.A.C. Bertollo https://orcid.org/0000-0001-6487-2834

W.F. Molina https://orcid.org/0000-0002-6695-0952

### Data availability

The data that support the findings of this study are available from the corresponding author upon reasonable request.
